# The emerging roles of Shank3 in cardiac function and dysfunction

**DOI:** 10.3389/fcell.2023.1191369

**Published:** 2023-04-28

**Authors:** Yoonhee Kim, Tae Hee Ko, Chunmei Jin, Yinhua Zhang, Hyae Rim Kang, Ruiying Ma, Huiling Li, Jong-Il Choi, Kihoon Han

**Affiliations:** ^1^ Department of Neuroscience, Korea University College of Medicine, Seoul, Republic of Korea; ^2^ Division of Cardiology, Department of Internal Medicine, Korea University College of Medicine and Korea University Anam Hospital, Seoul, Republic of Korea; ^3^ Center for Synaptic Brain Dysfunctions, Institute for Basic Science, Daejeon, Republic of Korea; ^4^ BK21 Graduate Program, Department of Biomedical Sciences, Korea University College of Medicine, Seoul, Republic of Korea

**Keywords:** Shank3, heart, cardiomyocyte, calcium homeostasis, mitochondria

## Abstract

Shank3 is a member of the Shank family proteins (Shank1–3), which are abundantly present in the postsynaptic density (PSD) of neuronal excitatory synapses. As a core scaffold in the PSD, Shank3 plays a critical role in organizing the macromolecular complex, ensuring proper synaptic development and function. Clinically, various mutations of the *SHANK3* gene are causally associated with brain disorders such as autism spectrum disorders and schizophrenia. However, recent *in vitro* and *in vivo* functional studies and expression profiling in various tissues and cell types suggest that Shank3 also plays a role in cardiac function and dysfunction. For example, Shank3 interacts with phospholipase Cβ1b (PLCβ1b) in cardiomyocytes, regulating its localization to the sarcolemma and its role in mediating Gq-induced signaling. In addition, changes in cardiac morphology and function associated with myocardial infarction and aging have been investigated in a few *Shank3* mutant mouse models. This review highlights these results and potential underlying mechanisms, and predicts additional molecular functions of Shank3 based on its protein interactors in the PSD, which are also highly expressed and function in the heart. Finally, we provide perspectives and possible directions for future studies to better understand the roles of Shank3 in the heart.

## Introduction

The SH3 and multiple ankyrin repeat domains gene family (*SHANK1*, *SHANK2*, and *SHANK3*) encode multi-domain scaffolding proteins that exist abundantly in the postsynaptic density (PSD) of neuronal excitatory synapses (Boeckers et al., 1999; Naisbitt et al., 1999; Sheng and Kim, 2000). As core scaffolds in the PSD, Shank proteins directly and indirectly interact with hundreds of other synaptic proteins with diverse functions, thereby organizing the macromolecular protein complex and regulating proper synaptic development and function (Sheng and Kim, 2011). Consistent with their critical roles in the PSD, many different types of variants (i.e., deletions, duplications, and missense/nonsense mutations) of all *SHANK* gene members have been identified in individuals diagnosed with numerous brain disorders, including autism spectrum disorders, intellectual disability, schizophrenia, and bipolar disorder (Grabrucker et al., 2011; Guilmatre et al., 2014; Ey et al., 2020). Accordingly, for the last decade, tens of different animal models carrying *Shank* gene mutations have been generated and investigated at the molecular, cellular, circuit, and behavioral levels, which greatly advanced our understandings on the brain mechanisms for the “Shankopathies”. For a comprehensive overview of the animal models of Shankopathies and their specific phenotypes, we recommend consulting expert reviews (Jiang and Ehlers, 2013; Yoo et al., 2014; Kazdoba et al., 2016; Monteiro and Feng, 2017; Eltokhi et al., 2018; Delling and Boeckers, 2021; Vyas et al., 2021; Jung and Park, 2022).

Clinically, individuals with *SHANK* gene mutations often present comorbidities associated with dysfunction of organs beyond the brain (Guilmatre et al., 2014; Leblond et al., 2014; Ricciardello et al., 2021). For instance, a significant portion of individuals with Phelan-McDermid syndrome (PMDS), who have *SHANK3* gene deletions, exhibit gastrointestinal abnormalities alongside neurodevelopmental, neurological, and psychiatric symptoms (Samogy-Costa et al., 2019; Levy et al., 2022). Moreover, studies on *Shank3* knock-out mice have revealed altered intestinal morphology and microbiota composition, which could be attributed to the loss of Shank3 in the intestinal epithelium (Pfaender et al., 2017; Sauer et al., 2019). Recent research has also shown the localization of Shank3 protein in Z-discs in the skeletal muscle sarcomere (Lutz et al., 2020). Loss of Shank3 resulted in shortened Z-discs and impairment of acetylcholine receptor clustering at the neuromuscular junctions of both *Shank3* knock-out mice and PMDS human induced pluripotent stem cell (hiPSC)-derived myogenic cells, which may contribute to the skeletal muscle hypotonia observed in PMDS patients (Lutz et al., 2020). Therefore, investigating the expression and function of Shank proteins in organs other than the brain could provide further insights into the mechanisms underlying the full clinical manifestations of Shankopathies.

In this review, we focus on the role of Shank3 proteins in the heart. We highlight their emerging significance in molecular pathways that maintain normal cardiac function, including calcium homeostasis, as well as their contribution to the pathogenesis of cardiac dysfunction associated with myocardial infarction and aging. Additionally, we discuss the remaining questions and perspectives necessary to advance our understanding of the complex interplay between Shank3 and other proteins in cardiomyocytes, such as cytoskeletal proteins.

### Shank3 expression in the heart

Traditional Northern blot analyses conducted on various human and rodent tissue samples have demonstrated a high level of Shank3 mRNA expression in the heart, which is comparable to, or even higher than the level in the brain (Lim et al., 1999; Wilson et al., 2003). Consistently, bulk tissue RNA sequencing (RNA-seq) datasets available on The Human Protein Atlas (https://www.proteinatlas.org/ENSG00000251322-SHANK3/tissue) indicate “heart muscle” is one of the top-ranked tissues expressing Shank3 mRNA (ninth out of the 54 tissues listed in the consensus dataset). The heart is a complex organ comprising four morphologically and functionally distinct chambers, and is composed of various cell types, such as cardiomyocytes, endothelial cells, fibroblasts, smooth muscle cells, pericytes, and immune cells (Litvinukova et al., 2020). According to the single-cell RNA-seq dataset for heart muscle on The Human Protein Atlas (https://www.proteinatlas.org/ENSG00000251322-SHANK3/single+cell+type/heart+muscle), Shank3 mRNA is predominantly detected in endothelial cells, followed by cardiomyocytes, smooth muscle cells, and fibroblasts, of heart muscle.

At the protein level, however, Shank3 in the heart muscle is indicated as “not detected” on The Human Protein Atlas (https://www.proteinatlas.org/ENSG00000251322-SHANK3/tissue/primary+data). Although it is not immediately clear why there is a discrepancy between mRNA and protein levels, we caution that protein expression analysis should be interpreted with care because the currently available Shank3 antibodies demonstrate varying performance depending on the analyses conducted (Lutz et al., 2022). Furthermore, several direct validations and functional analyses support the expression of Shank3 protein in the heart, especially in cardiomyocytes (as detailed below). However, since most of these studies have used rodent models, it is possible that Shank3 protein expression levels and distributions differ in human hearts. Therefore, further analysis is needed to confirm the expression of Shank3 proteins in human hearts.

**FIGURE 1:**
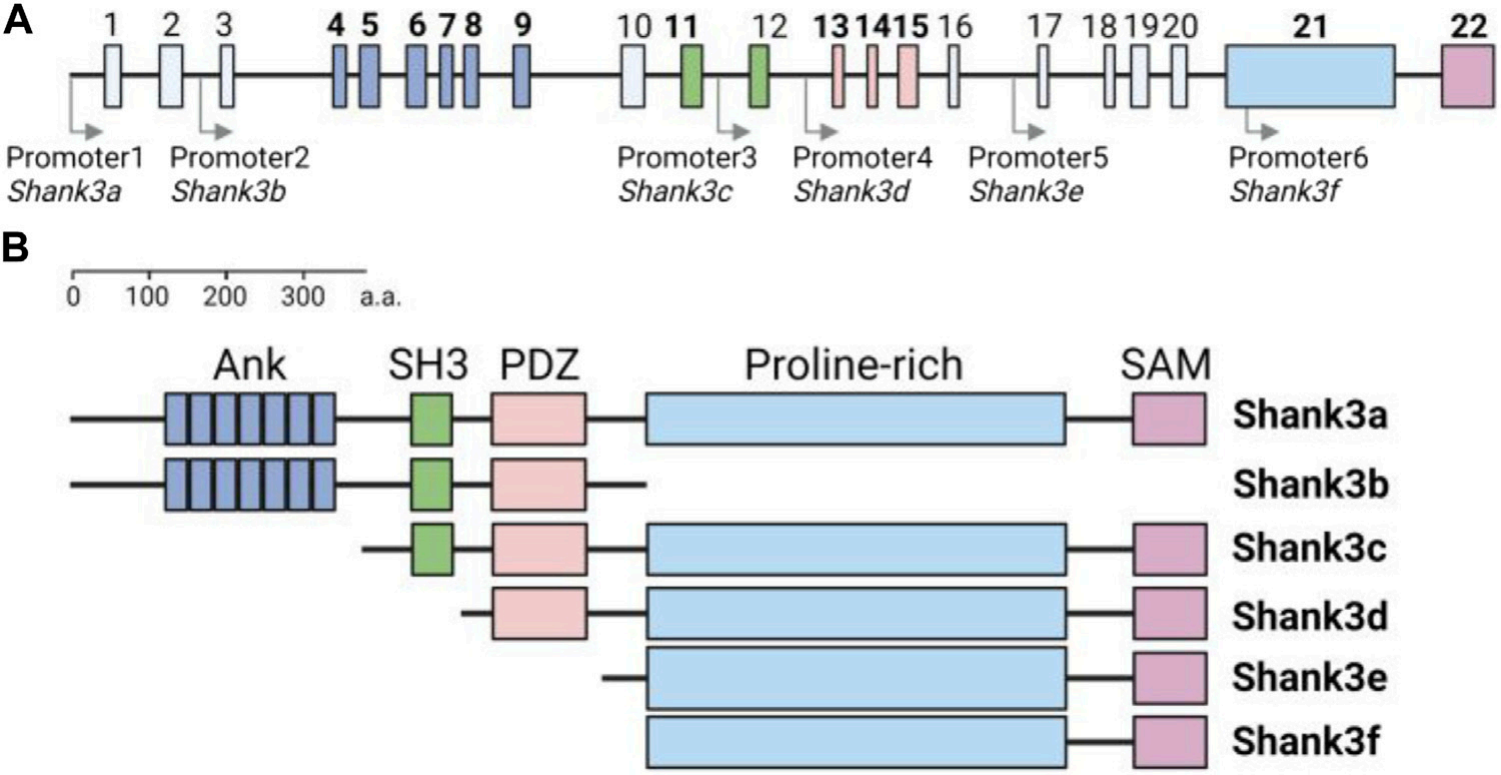
Structure of mouse *Shank3* gene and protein domain composition of Shank3 isoforms **(A)** Schematic diagram illustrating the structure of the mouse *Shank3* gene, which consists of 22 exons and multiple intragenic promoters indicated by arrows. The exons encoding protein domains are highlighted in bold and differentiated by color (Ank, exon 4–9; SH3, exon 11–12; PDZ, exon 13–15; Proline-rich, exon 21; SAM, exon 22). **(B)** Domain compositions of six Shank3 isoforms (Shank3 a–f). ANK, ankyrin repeats; SH3, Src homology 3; PDZ, PSD-95, Dlg, and Zo-1; SAM, sterile alpha motif. The figure was created with BioRender.com.

Another issue regarding Shank3 expression in the heart is the lack of detailed information about specific isoforms. Both human *SHANK3* and rodent *Shank3* genes express various isoforms due to multiple intragenic promoters and alternative splicing (Jiang and Ehlers, 2013; Wang et al., 2014) (Figure 1). In the brain, each Shank3 isoform has a distinct regional expression pattern, contributing to the clinical and phenotypic heterogeneity observed in patients and animal models (Lee et al., 2017a; Jin et al., 2018; Yoo et al., 2022). Therefore, it is important to determine whether the heart tissue also exhibits differential expression of Shank3 isoforms depending on the chambers and cell types.

### Shank3 interaction with PLCβ1b and regulation of calcium homeostasis in cardiomyocytes

**FIGURE 2:**
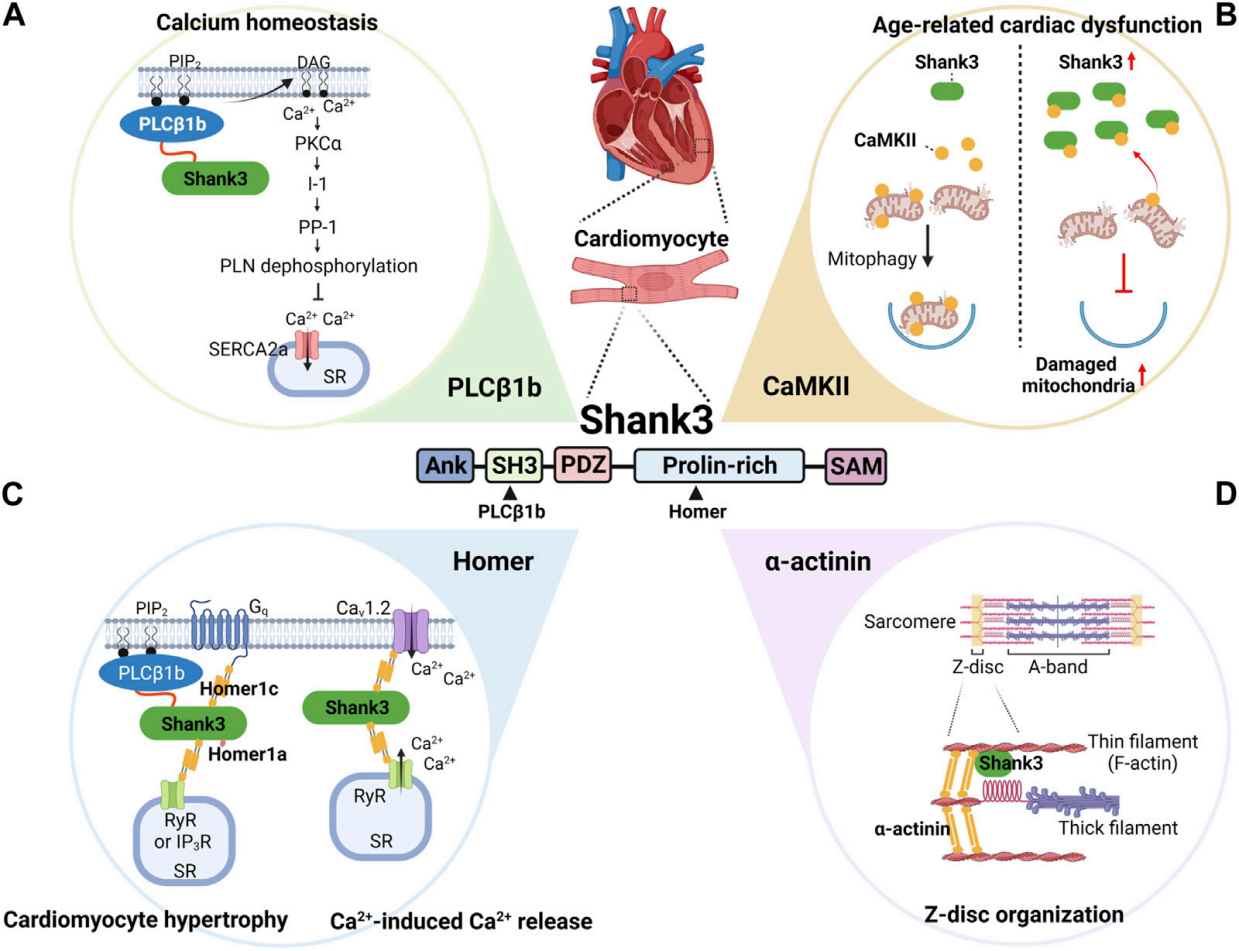
Summary of the demonstrated and predicted roles of Shank3 in cardiac function and dysfunction. In cardiomyocytes, Shank3 interacts with several proteins, including PLCβ1b **(A)**, CaMKII **(B)**, Homer **(C)**, and α-actinin **(D)**. Specifically, PLCβ1b and Homer bind to the SH3 and proline-rich domains of Shank3, respectively. By regulating the subcellular localization and activity of these interactors, Shank3 plays important roles in various physiological and pathological molecular pathways. These pathways include calcium homeostasis **(A)**, age-related cardiac dysfunction **(B)**, cardiomyocyte hypertrophy and calcium-induced calcium release **(C)**, and Z-disc organization **(D)**. ANK, ankyrin repeats; CaMKII, calcium-calmodulin-dependent protein kinase II; DAG, diacylglycerol; I-1, inhibitor-1; IP_3_R, inositol 1,4,5-triphosphate receptor; PDZ, PSD-95, Dlg, and Zo-1; PIP_2_, phosphatidylinositol 4,5-bisphosphate; PKCα, protein kinase Cα; PLCβ1b, phospholipase Cβ1b; PLN, phospholamban; PP-1, protein phosphatase-1; RyR, ryanodine receptor; SAM, sterile alpha motif; SERCA2a, sarco-endoplasmic reticulum ATPase 2a; SH3, Src homology 3; SR, sarcoplasmic reticulum. The figure was created with BioRender.com.

The first molecular function of Shank3 identified in cardiomyocytes was as an interaction partner of phospholipase Cβ1b (PLCβ1b) (Grubb et al., 2011) (Figure 2A). The C-terminal proline-rich sequences of PLCβ1b directly bind to the Src homology 3 (SH3) domain of Shank3 (Grubb et al., 2015), and as a result, PLCβ1b and Shank3 co-localize at the sarcolemma of cardiomyocytes.

In cardiomyocytes, PLCβ1b is activated by the Gq family of heterotrimeric G proteins, thus by G protein coupled receptors (GPCRs) such as *α*_1_-adrenergic receptors, and resulting in the hydrolysis of phosphatidylinositol 4,5-bisphosphate (PIP_2_) to generate inositol 1,4,5-triphosphate (IP_3_) and sn-1,2-diacylglycerol (DAG) (Filtz et al., 2009). This activation of Gq signals leads to cardiomyocyte hypertrophy, where PLCβ1b, but not its splice variant PLCβ1a, plays a critical downstream role in the process (Filtz et al., 2009). The functional difference between PLCβ1a and PLCβ1b can be attributed to their differential subcellular localizations; unlike PLCβ1b, PLCβ1a does not localize to the sarcolemma where PIP_2_ is concentrated in cardiomyocytes (Grubb et al., 2008).

The amino acid sequence difference between PLCβ1a and PLCβ1b is only in the C-terminal region. Specifically, PLCβ1a has a PSD-95, Dlg, and Zo-1 (PDZ)-binding motif in its C-terminal tail instead of proline-rich sequences, and thus cannot bind to the SH3 domain of Shank3. Therefore, the PLCβ1b-Shank3 interaction possibly explains the PLCβ1b-specific localization to the sarcolemma and its role in Gq-induced signaling to mediate cardiomyocyte hypertrophy. Supporting this notion, knock-down of *Shank3*, expression of full-length PLCβ1b with proline-to-alanine mutations in the C-terminal proline-rich sequences, and expression of only the C-terminal 32-aa sequences of PLCβ1b that possibly disrupt endogenous PLCβ1b-Shank3 interaction impair either PLCβ1b localization to the sarcolemma in cardiomyocytes or Gq-induced cardiomyocyte hypertrophy (Filtz et al., 2009; Grubb et al., 2011; Grubb et al., 2015).

In addition to regulating cardiomyocyte hypertrophy, PLCβ1b can also affect calcium homeostasis in cardiomyocytes by producing DAG (Figure 2A). DAG activates protein kinase Cα (PKCα), which in turn regulates the phosphorylation status of phospholamban (PLN) *via* the inhibitor-1 (I-1) and protein phosphatase-1 (PP-1) pathway (Woodcock and Grubb, 2015). Specifically, PKCα phosphorylates I-1, which activates PP-1 to dephosphorylate PLN. When PLN is dephosphorylated, it inhibits the sarco-endoplasmic reticulum ATPase 2a (SERCA2a), a pump critical for maintaining calcium homeostasis in cardiomyocytes (MacLennan and Kranias, 2003). SERCA2a transports calcium ions from the cytosol back to the sarcoplasmic reticulum (SR) during the cardiac contraction cycle. Dysregulation of SERCA2a can results in defects in intracellular calcium homeostasis, which is implicated in heart failure (Zhihao et al., 2020). Since the PLCβ1b-Shank3 interaction is critical for proper subcellular localization and activity of PLCβ1b in producing DAG, it is conceivable that Shank3 is involved in regulating calcium homeostasis in cardiomyocytes by indirectly regulating the activity of SERCA2a (Woodcock and Grubb, 2015).

### Cardiac function and dysfunction in *Shank3* mutant mice

To date, several *Shank3* mutant mouse models, including conventional and conditional knock-out, knock-in, and overexpression mice, have been generated and characterized (Jung and Park, 2022). Among them, various knock-out mouse models, which have different deletions of *Shank3* exons (out of total 22 exons in the mouse *Shank3* gene), exhibit both shared and distinct neurobehavioral phenotypes, possibly due to the deletion of specific Shank3 isoforms based on the affected exons (Jin et al., 2020; Delling and Boeckers, 2021) (Figure 1).

Taking into account this phenotypic heterogeneity, a recent study investigated the morphological and functional changes in the heart of adult *Shank3* exon four to nine heterozygous (*Δexon 4–9* ^*+/−*^) and homozygous (*Δexon 4–9* ^*−/−*^) knock-out mice (Assimopoulos et al., 2022). Since *Shank3* exon four to nine encodes the N-terminal ankyrin repeats (ANK) of Shank3, Shank3 isoforms containing SH3, PDZ, proline-rich, and the C-terminal sterile alpha motif (SAM) domains can still be normally expressed in the mice (Jiang and Ehlers, 2013). The *Shank3 Δexon 4–9* ^*+/−*^ mice displayed significantly increased left ventricular (LV) anterior and posterior wall thickness, while *Shank3 Δexon 4–9* ^*−/−*^ mice only displayed an increase in LV anterior wall thickness compared to wild-type mice (Assimopoulos et al., 2022). However, other cardiac parameters, including aorta diameter, heart rate, LV fractional shortening, stroke volume, and chamber diameter, remained normal in both *Shank3 Δexon 4–9* ^*+/−*^ and *Δexon 4–9* ^*−/−*^ mice.

Another study demonstrated the effects of Shank3 dosage on the morphological and functional changes in the heart after myocardial infarction (MI) by investigating the phenotypes in adult *Shank3* knock-out and overexpressing mice (Man et al., 2020). Unfortunately, detailed information, such as deleted *Shank3* exons and the promoter driving *Shank3* overexpression, about the *Shank3* knock-out and overexpressing mice, respectively, were not provided in the paper. Nevertheless, both morphological (infarct size) and functional (echocardiographic parameters, including LV ejection fraction and fractional shortening) changes after MI were significantly aggravated in *Shank3* knock-out mice, while they were alleviated in *Shank3* overexpressing mice. Mechanistically, after MI induction, increased apoptosis and decreased autophagy were observed in *Shank3* knock-out mice, while the opposite changes were seen in *Shank3* overexpressing mice compared to wild-type mice. The effects of Shank3 dosage on apoptosis and autophagy were replicated in primary neonatal cardiomyocytes after hypoxia condition (Man et al., 2020), confirming the protective role of Shank3 in cardiomyocytes after MI. The exact mechanism by which Shank3 regulates apoptosis and autophagy in cardiomyocytes remains unknown, but crosstalk between Shank3 and mitochondria (as detailed below) or the mechanistic target of rapamycin complex 1 (mTORC1) signaling (Bidinosti et al., 2016; Lee et al., 2017b), a negative regulator of autophagy (Kim and Guan, 2015), may be involved.

The role of Shank3 in age-related cardiac dysfunction has also been demonstrated in a study (Wang et al., 2022) (Figure 2B). This study found that aged (18-month-old) wild-type mice exhibited cardiac dysfunction, hypertrophy, and fibrosis along with increased cardiac Shank3 expression compared to young mice. Conversely, in cardiac-specific *Shank3* conditional knock-out (cardiac cKO) mice, which were generated by crossing *floxed-Shank3* mice and cardiac *α-myosin heavy chain-Cre* mice, age-related cardiac changes were alleviated. This indicates that increased Shank3 expression contributes to age-related cardiac dysfunction. At the molecular and cellular level, cardiomyocytes in *Shank3* cardiac cKO mice exhibited suppressed age-related increase in mitochondrial oxidative stress and apoptosis, and age-related decrease in mitophagy. Mechanistically, Shank3 interacts with calcium-calmodulin-dependent protein kinase II (CaMKII) and inhibits its translocation to mitochondria, which is enhanced in the aged heart as Shank3 expression increases. Reduced CaMKII translocation to mitochondria subsequently inhibited Parkin phosphorylation and translocation to mitochondria, critical steps in mitophagy activation (Vives-Bauza et al., 2010). Therefore, increase of Shank3 expression in the aged heart inhibits mitophagy in a CaMKII-dependent manner (Wang et al., 2022). These results somewhat contrast with the protective role of Shank3 in MI-induced cardiac dysfunction (Man et al., 2020). This difference in findings may be attributed to the different mouse models used (adult conventional vs. aged conditional knock-out) and/or the experimental setting (MI vs. aging).

### Prediction of additional molecular functions of Shank3 in cardiomyocytes

Numerous Shank3 interactors in the PSD have been identified using candidate-based and unbiased approaches, such as yeast two-hybrid screening and mass spectrometry-based proteomic analysis, followed by functional validation studies (Sakai et al., 2011; Han et al., 2013; Wang et al., 2020). Some of these synaptic interactors are also highly expressed in the heart and have already been shown to play essential roles in proper cardiac function. Specifically, we observed that 297 proteins from a recent Shank3 interactome (Wang et al., 2020) were present in at least one of three cardiac expression datasets (Supplementary Table S1). Therefore, in addition to the functions described above, it is possible that Shank3 can exert further molecular functions by interacting with and regulating these proteins in cardiomyocytes.

The Homer family proteins, comprising Homer1–3, act as scaffolds and well-established interactors of Shank3 in the PSD, where they together form a highly-organized lattice-like structure (Hayashi et al., 2009; Sheng and Kim, 2011). Moreover, Homer interacts with and regulates the activity and subcellular localization of several calcium-regulatory proteins, such as L-type calcium channels, ryanodine receptors (RyRs), IP_3_ receptors (IP_3_R), PLCβ, and transient receptor potential canonical (TRPC) channels, in various cell types (Worley et al., 2007). These interactions, including the Homer-Shank3 interaction, are mediated by the N-terminal Ena/VASP homology1 (EVH1) domain of Homer, which recognizes the proline-rich motifs in the binding partners (Supplementary Figure S1A). Long Homer proteins (the Homer1b/c isoforms, Homer2, and Homer3), but not short Homer1a, also contain a coiled-coil domain at the C-terminal region, which mediates the formation of tetrameric Homer (Hayashi et al., 2009) (Supplementary Figure S1B). Therefore, Homer can organize cellular microdomains through its interactions with calcium regulators and its multimerization, to efficiently and tightly regulate intracellular calcium signaling (Figure 2C). One example of such regulation in muscle cells, including cardiomyocytes, is the Homer1-dependent regulation of the excitation-contraction coupling by forming the Ca_v_1.2-Homer1-RyR2 complex (Worley et al., 2007). Homer1b/c solidifies the complex, while Homer1a relaxes it, thereby controlling the membrane depolarization-induced activation of the calcium-induced calcium release (Huang et al., 2007). It remains to be further investigated whether and how Shank3 is involved in Homer1-dependent regulation of calcium signaling in cardiomyocytes. A previous study has shown that Homer1c interacts with PLCβ1b and Shank3 in cardiomyocytes, mediating Gq-induced cardiomyocyte hypertrophy (Grubb et al., 2012).

Shank3 interacts with numerous actin-binding and -regulatory proteins in the PSD (Han et al., 2013; Wang et al., 2020), and Shank3 can also directly bind to actin through its N-terminal domain (Salomaa et al., 2021). Consistently, abnormalities in the synaptic actin cytoskeleton are considered to be the primary molecular mechanisms underlying the synaptic and behavioral deficits observed in *Shank3* mutant mice (Duffney et al., 2015). In cardiomyocytes, the F-actin filament, also known as the thin filament, is the key component that mediates the contractile function, and many actin-binding proteins, such as α-actinin, are concentrated at the Z-disc where one end of thin filament is anchored (Sequeira et al., 2014). In the skeletal muscle sarcomere, Shank3 interacts with α-actinin and co-localizes in Z-discs, and loss of Shank3 resulted in shortened Z-discs (Lutz et al., 2020). Therefore, Shank3 may have similar roles in cardiomyocytes, organizing the Z-disc by interacting with actin, α-actinin, and other actin-binding proteins (Figure 2D).

## Conclusion and perspectives

As we highlighted in this review, there is a growing body of evidence indicating the involvement of Shank3 in cardiac function and dysfunction, which is mediated by its interactions with several proteins, including PLCβ1b, CaMKII, Homer, and α-actinin (Figure 2). However, a comprehensive understanding of the molecular functions of Shank3 in the heart remains elusive. Fortunately, researchers have accumulated a wealth of knowledge, techniques, and tools, such as a variety of mouse models, that have been successfully employed to investigate the synaptic function of Shank3 and can be used to advance our understanding of its cardiac function. Firstly, assuming that Shank3 also acts as a scaffold in cardiac cells, one obvious direction to better understand its function is to fully identify the cardiac protein interactors of Shank3 through mass spectrometry-based analysis, which has been successfully used to identify the Shank3 interactome in the brain. Secondly, the detailed spatiotemporal expression profiles of different Shank3 isoforms in different cardiac cell types need to be investigated at the protein level. Based on this information, the cardiac phenotypes of the various *Shank3* mutant mouse models currently available can be investigated and interpreted. Moreover, the generation and characterization of additional *Shank3* mouse models that selectively remove or overexpress Shank3 in specific cardiac cell types will be crucial for advancing our understanding of Shank3 function in the heart.

It is becoming increasingly clear that the heart is not simply an organ that supplies blood to the body, but rather has a complex and bidirectional relationship with the brain (‘brain-heart crosstalk’), affecting brain function and even behavior (Hsueh et al., 2023). Therefore, understanding the roles of Shank3 in cardiac function and dysfunction will have broad implications for both basic and clinical research. Ultimately, this research may lead to more efficient ways to treat patients with Shankopathies.
