# Supplementary figures and images for: The emerging roles of Shank3 in cardiac function and dysfunction
Source: Front Cell Dev Biol. 2023 Apr 28;11:1191369. doi: 10.3389/fcell.2023.1191369 (PMC10175600; doi:10.3389/fcell.2023.1191369)

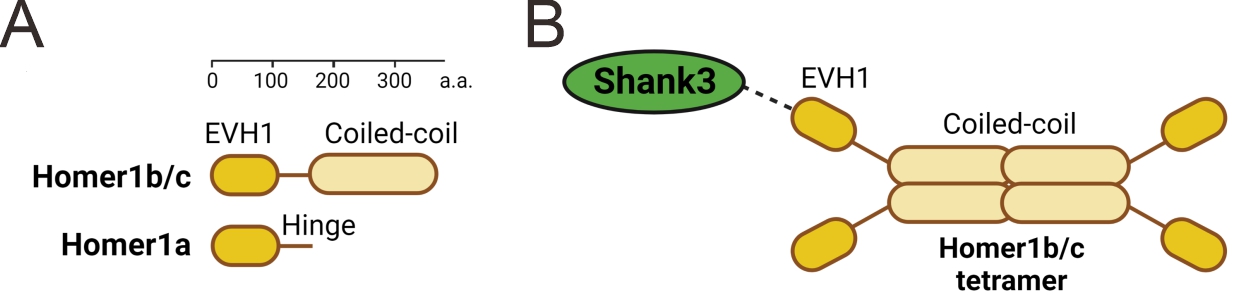

Supplement: Supplementary file 1 [file Image1.JPEG]
